# Characterization of a Novel Bat Adenovirus Isolated from Straw-Colored Fruit Bat (*Eidolon helvum*)

**DOI:** 10.3390/v9120371

**Published:** 2017-12-04

**Authors:** Hirohito Ogawa, Masahiro Kajihara, Naganori Nao, Asako Shigeno, Daisuke Fujikura, Bernard M. Hang’ombe, Aaron S. Mweene, Alisheke Mutemwa, David Squarre, Masao Yamada, Hideaki Higashi, Hirofumi Sawa, Ayato Takada

**Affiliations:** 1 Department of Virology, Okayama University Graduate School of Medicine, Dentistry and Pharmaceutical Sciences, 2-5-1 Shikata-cho, Kita-ku, Okayama 700-8558, Japan; masao@okayama-u.ac.jp; 2 Department of Disease Control, School of Veterinary Medicine, University of Zambia, P.O. Box 32379, Lusaka 10101, Zambia; asmweene04@yahoo.com (A.S.M.); hidea-hi@czc.hokudai.ac.jp (H.H.); h-sawa@czc.hokudai.ac.jp (H.S.); 3 Division of Global Epidemiology, Research Center for Zoonosis Control, Hokkaido University, N20, W10, Kita-ku, Sapporo 001-0020, Japan; kajihara@czc.hokudai.ac.jp (M.K.); n-nao@niid.go.jp (N.N.); shigeno@czc.hokudai.ac.jp (A.S.); 4 Division of Infection and Immunity, Research Center for Zoonosis Control, Hokkaido University, N20, W10, Kita-ku, Sapporo 001-0020, Japan; daikuke_fujikura@asahikawa-med.ac.jp; 5 Department of Paraclinical Studies, School of Veterinary Medicine, University of Zambia, P.O. Box 32379, Lusaka 10101, Zambia; mudenda68@yahoo.com; 6 Provincial Veterinary Office, Department of Veterinary Services, Ministry of Fisheries and Livestock, P.O. Box 70416, Ndola 50100, Zambia; malisheke@yahoo.com; 7 Department of National Parks and Wildlife, Ministry of Tourism and Arts, Private Bag 1, Chilanga 10101, Zambia; davidsquarre@yahoo.co.uk; 8 Hokudai Center for Zoonosis Control in Zambia, School of Veterinary Medicine, University of Zambia, P.O. Box 32379, Lusaka 10101, Zambia; 9 Global Institution for Collaborative Research and Education (GI-CoRE), Hokkaido University, N20, W10, Kita-ku, Sapporo 001-0020, Japan; 10 Division of Molecular Pathobiology, Research Center for Zoonosis Control, Hokkaido University, N20, W10, Kita-ku, Sapporo 001-0020, Japan; 11 Global Virus Network, 801 W Baltimore St, Baltimore, MD 21201, USA

**Keywords:** adenovirus, bat, *Eidolon helvum*, Zambia

## Abstract

Bats are important reservoirs for emerging zoonotic viruses. For extensive surveys of potential pathogens in straw-colored fruit bats (*Eidolon helvum*) in Zambia, a total of 107 spleen samples of *E. helvum* in 2006 were inoculated onto Vero E6 cells. The cell culture inoculated with one of the samples (ZFB06-106) exhibited remarkable cytopathic changes. Based on the ultrastructural property in negative staining and cross-reactivity in immunofluorescence assays, the virus was suspected to be an adenovirus, and tentatively named *E. helvum* adenovirus 06-106 (EhAdV 06-106). Analysis of the full-length genome of 30,134 bp, determined by next-generation sequencing, showed the presence of 28 open reading frames. Phylogenetic analyses confirmed that EhAdV 06-106 represented a novel bat adenovirus species in the genus *Mastadenovirus*. The virus shared similar characteristics of low G + C contents with recently isolated members of species *Bat mastadenoviruses E*, *F* and *G*, from which EhAdV 06-106 diverged by more than 15% based on the distance matrix analysis of DNA polymerase amino acid sequences. According to the taxonomic criteria, we propose the tentative new species name “*Bat mastadenovirus H*”. Because EhAdV 06-106 exhibited a wide in vitro cell tropism, the virus might have a potential risk as an emerging virus through cross-species transmission.

## 1. Introduction

In recent decades, bats have been recognized as an important carrier or potential carrier of emerging zoonotic viruses worldwide [1]. In Africa, fruit bats are a suspected reservoir of many emerging and re-emerging viruses, including filoviruses, coronaviruses, paramyxoviruses, and lyssaviruses [2]. Therefore, extensive surveys of potential pathogens in bats are essential to forestall outbreaks of emerging and re-emerging infectious diseases. Straw-colored fruit bats (*Eidolon helvum*) in Zambia, which are known to migrate from the Democratic Republic of Congo, are suspected to be a reservoir of several zoonotic pathogens [3,4,5], and have been extensively investigated in Zambia since 2006. In the process of this investigation, we isolated a novel adenovirus (*E. helvum* adenovirus 06-106: EhAdV 06-106) from this fruit bat species.

Adenoviruses are non-enveloped, 70–90 nm icosahedral viruses that contain a linear, double-stranded DNA genome. The members of the family *Adenoviridae* were classified into five genera, *Mastadenovirus*, *Aviadenovirus*, *Atadenovirus*, *Siadenovirus* and *Ichtadenovirus*, by the International Committee on Taxonomy of Viruses (ICTV) [6]. Human adenoviruses belonging to the genus *Mastadenovirus* were discovered first from human adenoid tissue and were classified into seven species, *Human mastadenovirus A* to *G* (HAdV-A to -G) [6,7]. Among animal adenoviruses in *Mastadenovirus*, two canine adenoviruses are well characterized. Canine adenoviruses 1 and 2 are causative agents of severe infectious hepatitis and common respiratory disease (infectious tracheobronchitis, known as kennel cough in puppies), respectively [8,9]. These two canine adenoviruses are classified into the single species *Canine mastadenovirus A* (CAdV-A). As the other animal adenoviruses in the genus *Mastadenovirus*, *Bovine mastadenovirus A* to *C* (BAdV-A to -C), *Equine mastadenovirus A* and *B* (EAdV-A and -B), *Murine mastadenovirus A* to *C* (MAdV-A to -C), *Ovine mastadenovirus A* and *B* (OAdV-A and -B), *Porcine mastadenovirus A* to *C* (PAdV-A to -C), *Simian mastadenovirus A* to *C* (SAdV-A to -C) and *Tree shrew mastadenovirus A* (TsAdV-A) are listed in the current ICTV database [10].

Since the first isolation of adenovirus from fruit bat (*Pteropus dasymallus yayeyamae*) in 2008 [11], several bat adenoviruses have been identified worldwide [12,13,14,15,16]. Bat adenovirus strains FBV1 [11], PPV1 [16] and TJM [14] are tentatively listed as bat adenoviruses-1 to -3 in order of the date they were reported. However, bat adenovirus 3 strain TJM [14] whose full-length genomic sequence was determined first, was approved as *Bat mastadenovirus A* (BtAdV-A) by ICTV [17]. Bat adenovirus 2 strain PPV1 was approved as *Bat mastadenovirus B* (BtAdV-B) based on the second full-length genomic sequence report [18]. These two prototype species BtAdV-A and -B (TJM and PPV1) are genetically closely related to CAdV-A.

Recently, three additional bat adenovirus strains (WIV9 to 11) were reported [19]. Interestingly, these viruses, which had long genes in their E3 region, were phylogenetically distant from CAdV-A and prototype species BtAdV-A and -B, and formed a second group of bat adenoviruses. In addition, four bat adenovirus strains (WIV12, 13, 17 and 18) showing low G + C content (31.3–34.4%) were discovered [20]. These strains were genetically closely related to the California sea lion adenovirus (CSLAdV-1) reported in 2015 [21], whose G + C content was also low (36.0%), and formed a third group.

In this study, we determined the full-length genomic sequence of the novel bat adenovirus (EhAdV 06-106) isolated from *E. helvum* captured in Zambia in 2006 and found that the virus also exhibited low G + C content and shared similar characteristics to bat adenoviruses in the third group. Its replication capacity was also estimated using cultured cell lines derived from various animal species. In addition, we implemented molecular epizootiology to identify related bat adenoviruses in this fruit bat species.

## 2. Materials and Methods

### 2.1. Animals and Samples

Tissue and DNA samples from wild straw-colored fruit bats (*Eidolon helvum*) captured in Kasanka National Park in the Central Province and in Ndola in the Copperbelt Province of Zambia were used. These samples were collected for our previous reports and stored at −30 °C or −80 °C [3,4,5]. All experiments were performed under the research project “Molecular and serological surveillance of viral zoonoses in Zambia” authorized by the Department of National Parks and Wildlife (DNPW) (formerly Zambia Wildlife Authority) of the Ministry of Tourism and Arts, Republic of Zambia. This study was performed with permission, following the guidelines (Act No. 12 of 1998).

### 2.2. Virus Isolation

For virus isolation, 10% (*w*/*v*) homogenates prepared from 107 spleen samples collected in 2006 were inoculated onto Vero E6 cells in 48-well plates (Corning, Corning, NY, USA). The plates were incubated for 1 h at 37 °C with 5% CO_2_ to permit adsorption on the virus. The cells were cultivated using fresh Eagle’s minimum essential medium (MEM) (Nissui Pharmaceutical Co., Tokyo, Japan) supplemented with 2% fetal calf serum (FCS) at 37 °C with 5% CO_2_ for 2 weeks or until cytopathic effect (CPE) appeared. The tissue culture (TC) supernatant was blind-passaged three times for monitoring of CPE.

For virus isolation from eight PCR-positive bats, 10% (*w*/*v*) homogenates from the spleen, liver and kidney samples per individual animal were processed and inoculated onto Vero E6 and Madin-Darby canine kidney (MDCK) cells according to the above protocol.

### 2.3. Transmission Electron Microscopy

Transmission electron microscopy was performed to observe the virion in the cultural supernatant. Virus samples were recovered from the TC supernatant in Vero E6 cells after centrifugation at 32,000 rpm at 4 °C for 3 h through a 20% sucrose cushion. A suspension fixed with 0.25% glutaraldehyde was adsorbed to collodion-carbon-coated copper grids (400 mesh; Nisshin EM Co., Tokyo, Japan) and negatively stained with 2% phosphotungstic acid (pH 5.8). The sample was observed with an H-7650 electron microscope (Hitachi High-Technologies, Tokyo, Japan) at 80 kV.

### 2.4. Indirect Immunofluorescence Assay

Vero E6 cells were infected with EhAdV 06-106 at a multiplicity of infection (MOI) of 1 and cultured for 16 h on 8-well chamber slides (Thermo Fisher Scientific, Waltham, MA, USA). After fixation with 4% paraformaldehyde for 30 min and permeabilization with 0.05% Triton X-100 for 10 min, the cells were incubated with anti-adenovirus type 5 polyclonal antibody (ab6982; Abcam, Cambridge, UK) diluted 1:800 in phosphate buffered saline (PBS) for 1 h at room temperature. The first antibody was detected with Alexa Fluor 488-conjugated goat anti-rabbit immunoglobulin G (IgG) (A11008; Invitrogen, Carlsbad, CA, USA) diluted 1:1000 in PBS for 1 h at room temperature. Fluorescent images were acquired using an LSM 780 confocal laser scanning microscope (Zeiss, Oberkochen, Germany).

### 2.5. Virus Purification and DNA Extraction for Next-Generation Sequencing

The virus was inoculated onto confluent Vero E6 cells in a T225 tissue culture flask (Corning, Corning, NY, USA), and the cells were maintained in MEM supplemented with 2% FCS at 37 °C with 5% CO_2_ for 5 days until CPE was observed in more than 70% of cells. The infectious supernatant and collected cells were freeze-thawed three times, then centrifuged at 5000 rpm at 4 °C for 30 min, and the cell debris was removed. The supernatant was centrifuged at 27,000 rpm at 4 °C for 2 h by the cesium chloride (CsCl) density gradient method using 2 M- and 4 M-CsCl in 10 mM 4-(2-hydroxyethyl)-1-piperazineethanesulfonic acid (HEPES) solution (pH 8.0), followed by collection of the viral particle phase. The collected viral solution was mixed with 10 mM HEPES solution (pH 8.0) and centrifuged at 27,000 rpm at 4 °C for 2 h. Viral DNA was extracted from the pellet with a Trizol LS (Thermo Fisher Scientific, Waltham, MA, USA) according to the manufacturer’s instructions after removing the supernatant.

### 2.6. Library Preparation and Full-Length Genomic Sequencing

Extracted viral DNA (100 ng) was tagged with index adaptors by using a TruSeq Nano DNA Library Prep kit (Illumina, San Diego, CA, USA) according to the manufacturer’s instructions. The 300-bp paired-end sequencing on a MiSeq instrument (Illumina, San Diego, CA, USA) was performed as described previously [22]. After sequencing, reads were subjected to de novo assembly by using CLC Genomics Workbench 7.5.1 software (CLC Bio, Aarhus, Denmark). Reads were analyzed with default settings, except for trimming (Q-score: >20; read length: >300 bp). The ORFs were searched using an Open Reading Frame Finder (http://www.ncbi.nlm.nih.gov/orffinder/) and then homologues were analyzed using a BLAST search.

### 2.7. Phylogenetic Analysis and Other Bioinformatics Analysis

Maximum-likelihood phylogenetic trees were constructed using MEGA 6.0 software [23]. The models to construct trees based on the nucleotide sequence and amino acid sequence were chosen from the lowest Bayesian information criterion in the Maximum-likelihood fits of 24 and 48 different substitution models, respectively. Adenoviruses used in the phylogenetic analysis based on the full-length genome and their accession numbers are listed in Table S1.

GENETYX-MAC Network version 18 (Genetyx, Tokyo, Japan) was used for global homology analysis. Full-length genome homologies between EhAdV 06-106 and bat adenovirus WIV17, as well as between EhAdV 06-106 and bat adenovirus WIV18, were compared by global genome pairwise using mVISTA [24]. Splice sites were predicted by the Genie program [25]. Distance matrix analysis of the DNA polymerase (pol) amino acid sequence was performed by using MEGA 6.0 software.

### 2.8. Analysis of In Vitro Cell Tropism of EhAdV 06-106

The animal cell lines Vero E6 (monkey kidney), ZFBK13-76E (*E. helvum* kidney), MDCK (dog kidney), PK-15 (pig kidney), RK-13 (rabbit kidney) and BHK-21 (hamster kidney), and HEK293T (human kidney) were grown in Dulbecco’s modified MEM (Sigma-Aldrich, St. Louis, MO, USA) supplemented with 10% FCS in 24-well plates at 37 °C with 5% CO_2_ for 1 day. ZFBK13-76E was established by transfection of a plasmid encoding the Simian virus 40 large T gene as described previously [26]. Subconfluent cultures of the cells grown in 24-well plates were inoculated with EhAdV 06-106 at an MOI of 0.01. Cells were incubated for 7 days, and susceptibility to the virus was evaluated by the appearance of CPE and the quantitation of viral genome copy numbers in the cultural supernatant by a SYBR Green I-based real-time PCR assay developed for detection of the EhAdV genome. Briefly, DNA was extracted with a QIAamp DNA Mini Kit (QIAGEN, Hilden, Germany) according to the manufacturer’s instructions from supernatant collected at 1–7 day(s) post-infection (dpi). The real-time PCR was performed using a StepOnePlus real-time PCR system (Applied Biosystems, Foster City, CA, USA) in a 10-μL reaction mixture containing 1 μL of extracted DNA, 300 nM of each primer to detect the *fiber* gene (EhAdV-fiber-qF1768 (5′-GAGTTGGTCCCACAGTTCTTG-3′) and EhAdV-fiber-qR1871 (5′-ATCAAAGTGTAGCGCACATACC-3′) designed in this study), and 5 μL of PowerUp SYBR Green Master Mix (Applied Biosystems, Foster City, CA, USA). The cycling protocol comprised 2 min of incubation at 50 °C, 2 min of incubation at 95 °C, followed by 40 cycles of 95 °C for 3 s and 60 °C for 30 s. PCR products were confirmed by melting curve analysis.

### 2.9. Nested PCR for Molecular Epizootiology and Direct Sequencing

A total of 365 *E. helvum* kidney DNA samples collected over the years 2010 to 2013, which were analyzed in a previous study [4] and stored at −30 °C, were screened for the bat adenovirus *pol* gene by nested PCR with TaKaRa Ex Taq Hot Start Version (TaKaRa Bio, Shiga, Japan) according to the manufacturer’s instructions. We used the primer sets described previously [14]. In the first PCR, a total volume of 25 μL reaction mixture containing 1 μL of DNA was amplified with pol-F and pol-R. One microliter of the first PCR product was used for the second PCR with pol-nF and pol-nR. The PCR program consisted of primary denaturation at 98 °C for 1 min, followed by 30 cycles of denaturation at 98 °C for 10 s, annealing at 48 °C for 30 s, extension at 72 °C for 30 s, and final extension at 72 °C for 5 min.

Positive products of the second PCR were purified and subjected to direct sequencing using a BigDye Terminator v3.2 Cycle Sequencing Kit (Thermo Fisher Scientific, Waltham, MA, USA) according to the manufacturer’s instructions, and a 3130 Genetic Analyzer (Applied Biosystems, Foster City, CA, USA).

### 2.10. Analysis of the 5′ End of the Pol mRNA

Vero E6 cells in 6-well plates were infected with EhAdV 06-106 at an MOI of 1 and cultured for 2 days at 37 °C with 5% CO_2_. Total RNA was extracted from virus-infected cells by using NucleoSpin RNA (MACHEREY-NAGEL, Düren, Germany) according to the manufacturer’s instructions. Then, 1.5 μg total RNA was subjected to analysis with a 5′-Full RACE Core Set (TaKaRa Bio, Shiga, Japan) and KOD-Plus-Neo (TOYOBO, Osaka, Japan) with primers targeting a 5′ region of the *pol* gene (primer sequences are available upon request) according to the manufacturer’s instructions.

The PCR products were ligated into the pGEM-T Easy Vector (Promega, Madison, WI, USA) and transformed into ECOS Competent *E. coli* DH5α (NIPPON GENE, Tokyo, Japan). Plasmid DNA was purified from the cells with NucleoSpin Plasmid EasyPure (MACHEREY-NAGEL, Düren, Germany) according to the manufacturer’s instructions, and its insertion was confirmed by PCR using primers designed for the vector sequence. The plasmid DNA was then sequenced.

## 3. Results

### 3.1. Isolation of a Novel Adenovirus from E. helvum Captured in Zambia

**Figure 1:**
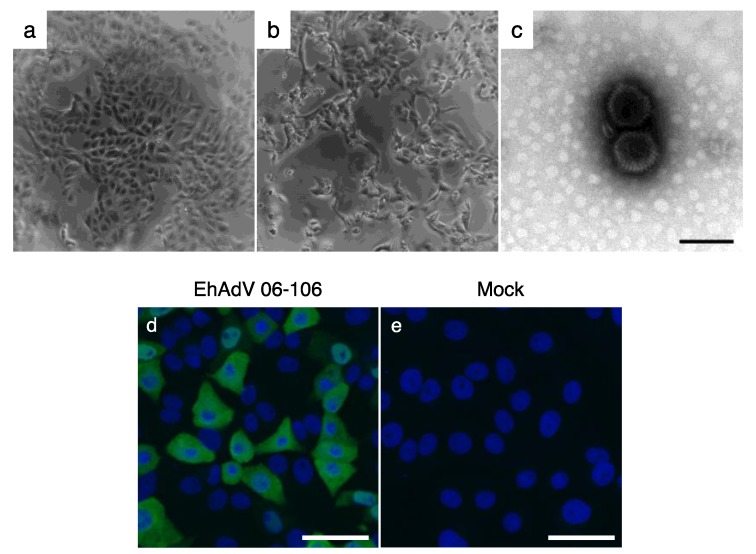
Isolation of *E. helvum* adenovirus (EhAdV) 06-106 with Vero E6 cells (upper panels). Control cells (**a**) and infected cells showing cytopathic effects (**b**); Transmission electron microgram of EhAdV 06-106 virions (**c**); Bar represents 100 nm. Detection of viral antigens in EhAdV 06-106-infected Vero E6 cells using anti-human adenovirus type 5 polyclonal antibody (lower panels). EhAdV 06-106-infected cells (**d**) and mock-infected cells (**e**); Nuclei of cells were stained with DAPI (4′,6-diamidino-2-phenylindole) (blue). Scale bars represent 50 μm.

CPE was observed in Vero E6 cells inoculated with homogenates from a spleen sample (ZFB06-106) at the second passage (Figure 1a,b). The TC supernatant of this well was expanded using Vero E6 cells and the virus stock was prepared for further characterization. We first confirmed the presence of virions in the supernatant of the infected cells using transmission electron microscopy. Approximately 80 nm non-enveloped icosahedral virus particles, compatible to the characteristic ultrastructure of adenoviruses, were observed (Figure 1c). Next, an indirect immunofluorescent assay was performed using an anti-human adenovirus type 5 antibody and clear fluorescence was observed in infected cells (Figure 1d,e), confirming that the isolated virus was a member of the genus *Mastadenovirus*. Thus, the virus was tentatively named *E. helvum* adenovirus strain 06-106 (EhAdV 06-106).

### 3.2. Whole Genome Analysis of EhAdV 06-106

**Figure 2:**
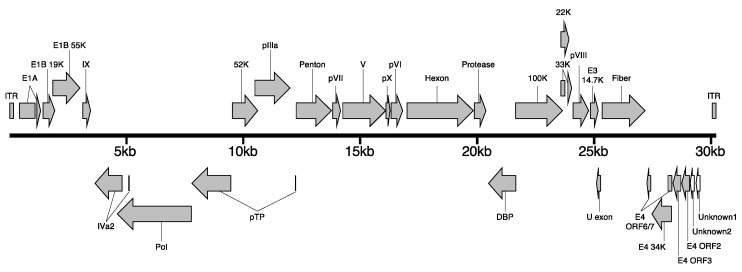
Genome organization of EhAdV 06-106. The genome of EhAdV 06-106 is represented by a bold bar, with the scale indicated below. Viral genes and inverted terminal repeats (ITR) sequences are shown as arrows or rectangles, respectively. The gray-shaded arrows represent open reading frames (ORFs) homologous to reported bat adenoviruses; open arrows represent unknown ORFs. Coding regions linked with a line indicate the predicted spliced transcript.

We determined the full-length genome sequence of EhAdV 06-106 by next-generation sequencing. DNA was extracted from a virus suspension derived from the TC supernatant of infected Vero E6 cells, and the DNA library was generated for Illumina MiSeq (Illumina, San Diego, CA, USA). By de novo assembly of the obtained fragments (a total of 3,544,267 reads) using a CLC Genomics Workbench (CLC Bio, Aarhus, Denmark), we obtained 30,134 bp of the full-length genome sequence of EhAdV 06-106, including a 102 bp sequence of inverted terminal repeats (ITR). The full-length genome sequence of the virus has been deposited in the DNA Data Bank of Japan (DDBJ) data bank under accession number AP018374. EhAdV 06-106 has a G + C content of 35.2%, and its DNA base compositions are 33.2% A, 17.2% C, 18.0% G and 31.6% T. A total of 30 open reading frames (ORFs) were predicted, and 28 of these ORFs were analogous to previously reported bat adenovirus homologs (Figure 2).

**Figure 3:**
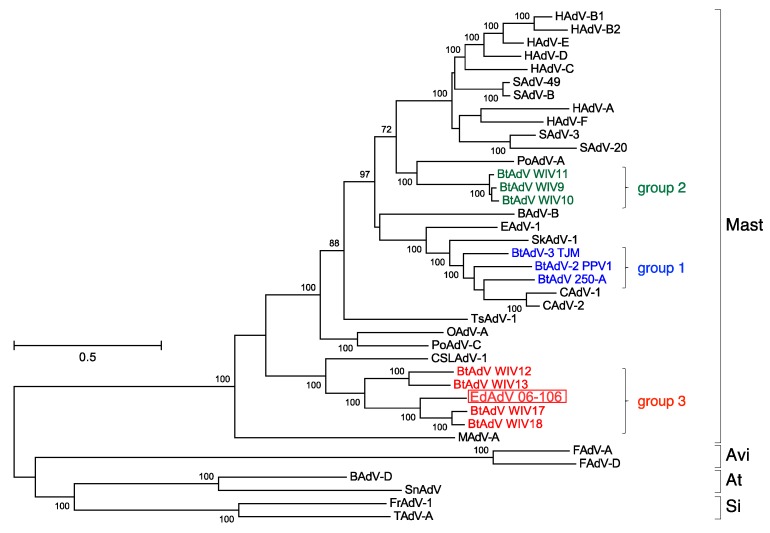
Maximum-likelihood phylogenetic trees based on the full-length of adenovirus genome. The dendrogram was constructed with the general time-reversible model with gamma distribution and invariable sites, with the highest log likelihood (−506,682.7635). The percentage at nodes are bootstrap probabilities that were calculated using 1000 replicates and indicate bootstrap supports >70%. Adenovirus genomes compared with EhAdV 06-106 were listed in Table S1. Bat adenoviruses are shown in colors. Open rectangle indicates EhAdV 06-106. Bar indicates the number of substitutions per site. Abbreviations: Mast, *Mastadenovirus*; Avi, *Aviadenovirus*; At, *Atadenovirus*; Si, *Siadenovirus*.

The phylogenetic tree, constructed by using full-length adenoviral genome sequences retrieved from GenBank, showed that EhAdV 06-106 belonged to the genus *Mastadenovirus* (Figure 3). In the phylogenetic tree, the bat adenoviruses were apparently divided into 3 groups: Group 1 including the ICTV-approved prototype species (BtAdV-A and -B) closely related to CAdV-A, group 2 having a long *E3* gene and group 3 having low G + C content (Table 1). Groups 2 and 3 formed separate clusters, both of which were placed distantly from group 1 and CAdV-A. EhAdV 06-106 was placed in the group 3 cluster and was most closely related to bat adenovirus strains WIV17 and WIV18 found in the fruit bat species *Rousettus leschenaultia* [20]. The amino acid identities in the 28 ORFs between EhAdV 06-106 and each of WIV17 and WIV18 ranged from 35.0 to 85.9% and 35.0 to 84.9%, respectively (Table 2). The amino acid similarity and identity between the *hexon* genes of EhAdV 06-106 and HAdV-C (human adenovirus type 5 [27]) were 88.2% and 63.5%, respectively, as was expected by cross-reactive immunofluorescence (Figure 1d).

In nucleotide levels, the coding regions of E1A, protein V (V; minor core protein), fiber and E4 unit sequences showed relatively lower identities (<50%) by global pairwise comparison between EhAdV 06-106 and bat adenovirus WIV17, as well as between EhAdV 06-106 and bat adenovirus WIV18 (Figure S1). The V (629 aa) sequence of EhAdV 06-106 was longer than those of the other bat adenoviruses, ranging from 364 aa in WIV12 to 582 aa in WIV17. In the EhAdV 06-106 E4 unit region, only four ORFs were predicted to be WIV17 and WIV18 homologues (E4 ORFs 6/7, 34K, 3 and 2). Alternatively, two unique ORFs (unknown1 with 75 aa and unknown2 with 42 aa) were predicted.

Next, we analyzed in silico whether pol and pre-terminal protein (pTP), both of which were related to viral DNA replication, were spliced from a common leader sequence that was predicted by sequence analysis in the previously reported adenoviruses [29]. In BtAdV-A and BtAdV-B, a 9 bp sequence including the initiation ATG codon supported the splicing of both the pol and pTP exons downstream. However, this 9 bp leader (ATGGCTTTG) of EhAdV 06-106 supported the splicing only to the pTP exon, but did not support splicing to the pol exon. The splicing of the *pol* mRNA using the predicted splicing donor and acceptor sites led to a stop codon. To confirm this, we extracted RNA from the EhAdV 06-106-infected Vero E6 cells and analyzed the 5′ end of the *pol* mRNA sequence. We obtained a non-spliced sequence, demonstrating that splicing did not occur in EhAdV 06-106. We found that this property was shared with other bat adenovirus strains WIV17 and WIV18 in group 3.

### 3.3. Phylogenetic Analyses of the Pol and Hexon Proteins

**Figure 4:**
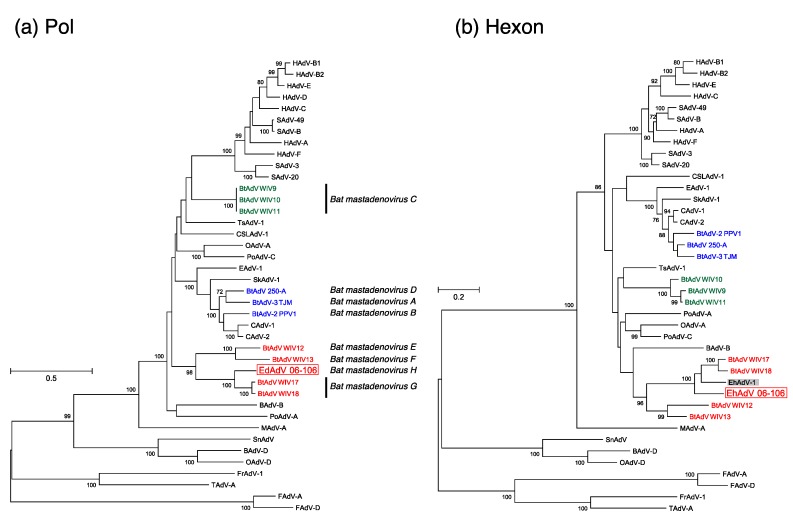
Maximum-likelihood phylogenetic trees based on the predicted amino acid sequences for DNA polymerase (pol) (**a**) and hexon (**b**). The dendrograms of pol and hexon were constructed using an LG (Le and Gascuel, 2008) model with gamma distribution and invariable sites with the highest log likelihood (−27,885.86709), and an LG model with gamma distribution with the highest log likelihood (−20,968.7769), respectively. The percentages at nodes are bootstrap probabilities calculated using 1000 replicates and indicate bootstrap supports >70%. Bat adenoviruses in groups 1 to 3 in Figure 3 are shown in colors. Open rectangles and gray-shaded rectangle indicate EhAdV 06-106 and EhAdV 1 isolated in Ghana, respectively. Bars indicate the number of substitutions per site. The sequences listed in Table S1 and EhAdV 1 (GenBank accession number: JX885602) were used.

The results of the phylogenetic analysis of the pol and hexon proteins based on the predicted amino acid sequences of EhAdV 06-106 are shown in Figure 3. In the phylogenetic tree based on the pol protein, EhAdV 06-106 was clustered together with bat adenovirus strains WIV17 and WIV18 (*Bat mastadenovirus G*) (Figure 4a). However, the phylogenetic distances between EhAdV 06-106 and bat adenovirus strains WIV17 and WIV18 were 15.68% and 15.55%, respectively. Since the phylogenetic distance based on the pol amino acid sequence using distance matrix analysis (>5–15%) is one of the species demarcation criteria in *Mastadenovirus* [6], our data support the proposal of a new species, *Bat mastadenovirus H*.

In order to compare the phylogenetic topology between EhAdV 06-106 and the first bat adenovirus isolated from *E. helvum* urine samples in Ghana and reported in 2013 (EhAdV 1) [30], for which only the hexon sequence was available in the database, we constructed a phylogenetic tree for the hexon amino acid sequence (Figure 4b). EhAdV 06-106 and EhAdV 1, as well as bat adenovirus strains WIV12, 13, 17 and 18, belonged within the sister cluster of BAdV-B [31]. The hexon amino acid sequence of EhAdV 06-106 shared 82.4% identity with that of EhAdV 1.

### 3.4. In Vitro Cell Tropism of EhAdV 06-106

**Figure 5:**
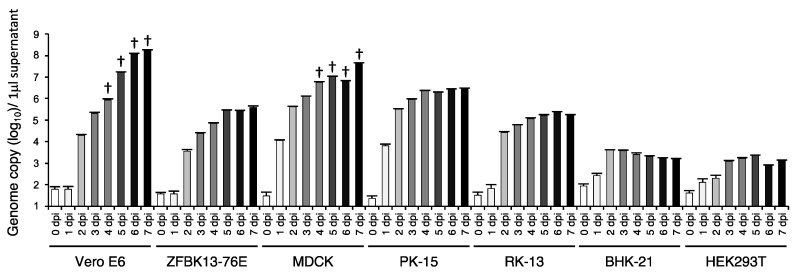
Quantitation of viral DNA in the supernatants of various mammalian cells infected with EhAdV 06-106 using real-time PCR. Data are shown as the means and standard deviations from triplicate reactions. Daggers indicate the points when cytopathic effect was remarkable. Dpi indicates day(s) post infection. ZFBK: Fruit bat kidney; MDCK: Madin-Darby canine kidney; PK: Porcine kidney; RK: Rabbit kidney; BHK; Baby hamster kidney; HEK: Human embryonic kidney.

To investigate the potential host range of EhAdV 06-106, seven different cell liens were inoculated with the virus. Interestingly, all cell lines were found to be susceptible to the virus (Figure 5) indicating that EhAdV 06-106 had a wide range of in vitro cell tropism. The virus replicated most efficiently in Vero E6 and MDCK cells, where it exhibited remarkable CPE at 4 dpi.

### 3.5. Prevalence of Bat Adenoviruses in Zambia

**Figure 6:**
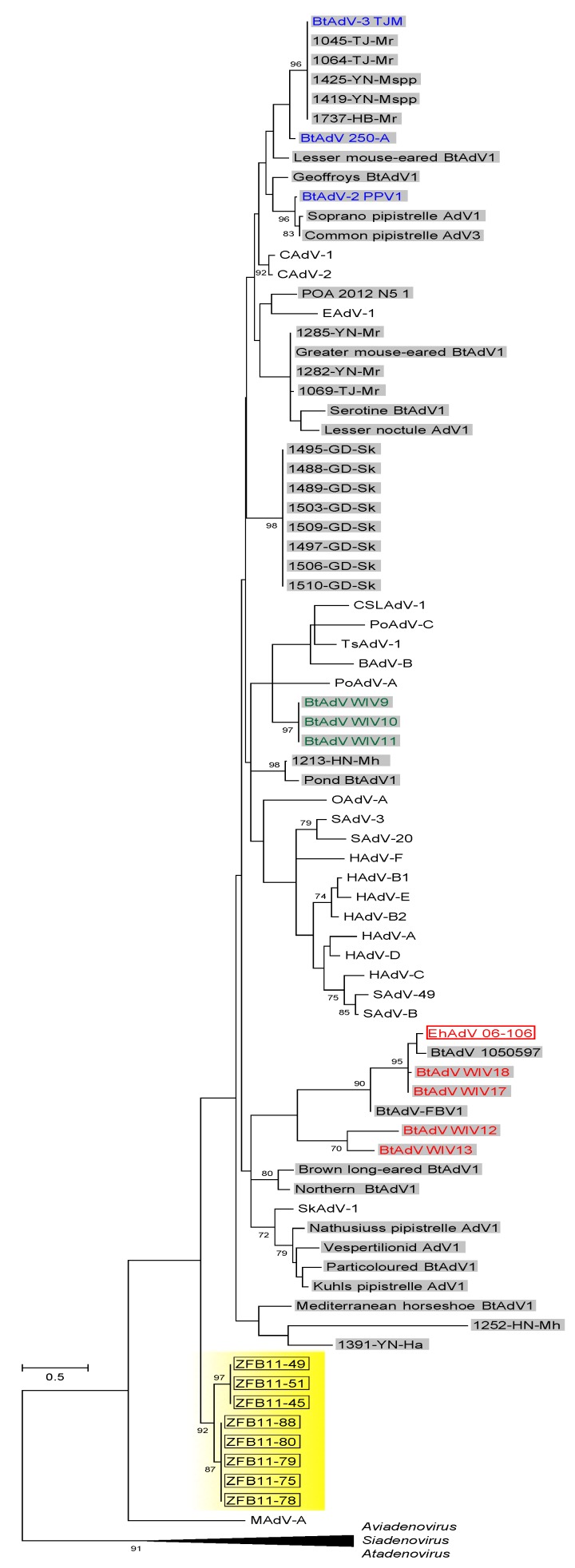
Maximum-likelihood phylogenetic trees based on the partial amino acid sequences of DNA polymerase (pol). The dendrogram was constructed with an LG model with gamma distribution with the highest log likelihood (−3122.742588). The percentages at nodes are bootstrap probabilities calculated using 1000 replicates and indicate bootstrap supports >70%. Open rectangles and gray-shaded rectangles indicate bat-derived adenoviruses detected in *E. helvum* in this study and bat-derived adenoviruses, respectively. Bat adenoviruses in groups 1 to 3 in Figure 3 are shown in colors. Eight newly detected *E. helvum*-derived adenoviruses are highlighted in yellow. Bar indicates the number of substitutions per site. The sequences listed in Table S1 and previously reported in bat adenoviruses [12,13].

The *pol* gene fragment was amplified from eight out of 365 kidney samples (2.19%), all of which were collected from bats captured in Kasanka National Park in 2011 (Table 3). The sequence of the eight PCR products (ZFB11-45, ZFB11-49, ZFB11-51, ZFB11-75, ZFB11-78, ZFB11-79, ZFB11-80 and ZFB11-88) were deposited to DDBJ and assigned to accession numbers LC324692 to LC324699. In the phylogenetic calculations based on the deduced amino acid sequences, these sequences formed a unique cluster distinct from all other bat adenoviruses including EhAdV 06-106 (Figure 6).

Our attempts to isolate infectious virus from any of the PCR-positive bats failed even after three blind passages. No CPE was visible, neither PCR-amplification of adenoviral DNA from the TC supernatant was successful.

## 4. Discussion

*E. helvum* bats are widely distributed in Africa because their prime habitat is the tropical forests of Central Africa, and from that centralized location they can easily migrate to the rest of the continent [32,33]. Over the past decade, bats have been extensively studied as an important reservoir and/or vector of emerging and re-emerging infectious agents. Indeed, we have demonstrated that *E. helvum* harbors several zoonotic pathogens in Zambia [3,4,5]. In this study, we isolated a novel bat adenovirus (EhAdV 06-106) from *E. helvum* in Zambia and characterized it genetically and biologically. In addition, we performed a molecular epizootiology study to find related bat adenoviruses in *E. helvum*.

A total of 11 full-length genome sequences of bat adenoviruses including EhAdV 06-106 have been made available to date. We analyzed these full-length genomes phylogenetically (Figure 3), and demonstrated that these bat adenoviruses clearly form three groups largely corresponding to their host family classification, i.e., group 1 contains viruses of members of the *Vespertilionidae*, group 2 those of *Rhinolophidae*. However, group 3 includes viruses of bats from two families *Miniopteridae* and *Pteropodidae* (Table 1). These latter families are of two different suborders (*Microchiroptera* and *Megachiroptera*, respectively). Interestingly, the groups 2 and 3 viruses, which were recently reported, formed separate clusters distinct from CAdV-A, while the group 1 viruses were closely related to CAdV-A. Based on the pol amino acid sequence that was a species demarcation criterion [6], the phylogenetic distances between EhAdV 06-106 and the two most closely related strains WIV17 and WIV18 were 15.68% and 15.55%, respectively (Figure 4a), suggesting that EhAdV 06-106 is likely to be a novel species, which we provisionally propose to be *Bat mastadenovirus H*. The first adenovirus derived from *E. helvum* bats (EhAdV 1) was identified in Ghana and reported in 2013 [30]. Although only the hexon sequence is available for this prototype virus, the amino acid identity of this region with EhAdV 06-106 is relatively high (82.4% identical), suggesting that EhAdV 06-106 or related adenoviruses might be commonly maintained in this bat species and distributed across Africa, since *E. helvum* bats are widely found in sub-Saharan Africa and capable of migrating thousands of kilometers across Central Africa [33].

Adenoviruses are believed to be co-evolved with their specific hosts, and usually infect one particular or several, closely related host species [29]. Indeed, viruses in the first two genera accepted taxonomically, *Mastadenovirus* and *Aviadenovirus*, are host-specific and thought to be highly adapted to some mammalian and avian species, respectively. In contrast, *Atadenovirus* and *Siadenovirus* were adopted as newer genera on the basis of the genome organization and characteristics of coding proteins, and viruses belonging to these genera have been detected in a relatively wide range of host species [6,34]. For example, *Atadenovirus* is so named because of the high A + T content in its genome, and its hosts include cattle, ducks, goats, possums and different squamate reptiles including snakes and lizards. Since the adenoviruses found in reptiles have equilibrated base composition, it has been hypothesized that the ancestral reptile adenovirus might have jumped to certain avian and mammalian hosts, where they further evolved [28,35]. The exact reason and mechanism behind the alteration of the base composition towards A + T bias, however, is not fully explored [36].

CAdV-A has also been hypothesized to have switched hosts because of its exceptional pathogenicity and wide host spectrum including numerous carnivorous animals. A bat adenovirus has been supposed to be the possible ancestor of CAdVs on the basis of their close genetic relatedness [18]. It is now presumed that a bat adenovirus jumped into carnivores (i.e., dogs) at some point in the past and evolved within this second host, resulting in the emergence of CAdV-A and the acquisition of its pathogenicity to a wide range of species [18,28]. Such host-jumping events are inferred from the close genetic link between prototype species (BtAdV-A and -B) and CAdV-A (Figure 3).

The discovery of new bat adenoviruses in group 3—including EhAdV 06-106 and WIV12, 13, 17 and 18 (Table 1)—suggests that cross-species transmissions might have occurred between bat species (i.e., *E. helvum*, *M. schreibersii* and *R. leschenaultii*). It is also worth noting that these newly found adenoviruses share characteristics similar to the above-mentioned *Atadenovirus*. The G + C contents of the group 3 viruses ranged from 31.3 to 35.2% (Table 1). It has been suggested that adenoviruses containing low genomic G + C contents have increased potential to transmit to other host species and adapt to these new hosts rapidly because low G + C contents are an efficient way to avoid the toll-like receptor 9-mediated innate immunity that recognizes unmethylated CpG dinucleotides [20,21,36,37]. Accordingly, we found that EhAdV 06-106 exhibited a broad range of in vitro cell tropism (Figure 5).

In our molecular epizootiological study, a 261-bp sequence of the *pol* gene was detected in 8 out of 365 (2.19%) *E. helvum* kidney samples collected during 2010–2013. The phylogenetic analysis revealed that these sequences formed a unique cluster that was clearly distinct from the cluster including EhAdV 06-106 and the other previously known bat adenoviruses (Figure 6), suggesting the existence of new bat adenoviruses circulating in this bat species. However, since virus isolation from the 8 PCR-positive bats was unsuccessful and the phylogenetic information that could be obtained from such short fragments of the *pol* gene was limited, the biological and genetic properties of these viruses remain unknown. Since this study was conducted as a part of the large-scale surveillance aiming at the clarification of the possible role of *E. helvum* bats in the maintenance and spreading of zoonotic pathogens [3,4,5], limited organs (i.e., spleen, liver and kidney) were available for the isolation and molecular detection of the bat adenovirus. Because high DNA copy numbers of a group 1 bat adenovirus (i.e., BtAdV-B) were detected in the intestine of the infected *P. pipistrellus* bats [16,18], it might also be interesting to use other organs (e.g., intestine) to isolate bat adenoviruses from *E. helvum* bats. We assume that several phylogenetically different adenoviruses were circulating among this bat species migrating in the Central and Southern African regions or a novel adenovirus was incidentally transmitted to *E. helvum* from other bat species in that particular year. Further studies are needed to clarify the whole picture of the ecology of bat adenoviruses in nature.

## Figures and Tables

**Table 1:** Grouping of 11 bat adenoviruses with reported full-length genome sequences and comparisons among the groups.

Group	Strain	ICTV Species *	Host	Country	Full-Genome (bp)	ITR (bp)	E3 Region		G + C Content (%)	Reference
							Product	Total Length (bp) ^†^		
Group 1	PPV1	*Bat mastadenovirus B*	*Pipistrellus pipistrellus*	Germany	31,616	137	12.5K, ORF1	1524	53.5	[18]
	TJM	*Bat mastadenovirus A*	*Myotis ricketti*	China	31,681	128	ORFA	1149	56.9	[14]
	250-A	(*Bat mastadenovirus D*)	*Corynorhinus rafinesquii*	United States	31,484	239	12.5K, ORFA	1515	49.8	[28]
Group 2	WIV9	(*Bat mastadenovirus C*)	*Rhinolophus sinicus*	China	37,545	35	12.5K, E3L, E3s	5145	55.5	[19]
	WIV10	(*Bat mastadenovirus C*)	*Rhinolophus sinicus*	China	37,556	51	12.5K, E3L, E3s	5133	55.2	[19]
	WIV11	(*Bat mastadenovirus C*)	*Rhinolophus sinicus*	China	38,073	51	12.5K, E3L, E3s	5631	55.0	[19]
Group 3	WIV12	(*Bat mastadenovirus E*)	*Miniopterus schreibersii*	China	29,581	73	14.7K	390	34.2	[20]
	WIV13	(*Bat mastadenovirus F*)	*Miniopterus schreibersii*	China	29,162	61	14.7K	363	31.3	[20]
	WIV17	(*Bat mastadenovirus G*)	*Rousettus Ieschenaultii*	China	29,923	178	14.7K	396	34.3	[20]
	WIV18	(*Bat mastadenovirus G*)	*Rousettus Ieschenaultii*	China	29,812	177	14.7K	396	34.2	[20]
	EhAdV 06-106	(*Bat mastadenovirus H*)	*Eidolon helvum*	Zambia	30,134	102	14.7K	378	35.2	This study

* Species given in parentheses indicate tentatively proposed species by the authors; ^†^ Total length of the protein-encoding genes. Abbreviations: International Committee on Taxonomy of Viruses, ICTV; Inverted terminal repeats, ITR; Open reading frame, ORF; *E. helvum* adenovirus, EhAdV.

**Table 2:** Genome components of EhAdV 06-106 and comparison with those of bat adenovirus strains WIV17 and WIV18.

	EhAdV 06-106			Bat Adenovirus WIV17		Bat Adenovirus WIV18	
Gene Product	Genomic Position *	Gene (nt)	Protein (AA)	Protein (AA)	Identity (%)	Protein (AA)	Identity (%)
ITR	1–102	102	N.A. ^†^	N.A.	N.A.	N.A.	N.A.
E1A	487–1090, 1230–1294	669	222	195	46.1	195	44.1
E1B small	1529–2014	486	161	168	53.4	168	44.7
E1B large	1909–3180	1272	423	425	59.5	425	60.2
IX	3240–3506	267	88	83	46.9	82	51.2
Iva2	3550–4874c, 5153–5165c	1338	445	422	82.7	422	82.7
Pol	4638–7895c	3258	1085	1086	80.4	1086	81.1
pTP	7892–9682c, 12,334–12,342c	1780	599	556	78.8	556	80.2
52K	9697–10821	1125	374	373	80.4	373	79.3
pIIIa	10,742–12,319	1578	525	521	82.8	521	82.4
Penton	12,377–13,768	1392	463	464	82.9	464	83.8
pVII	13,771–14,115	345	114	118	83.9	118	83.9
V	14,159–16,048	1890	629	582	63.6	571	65.9
pX	16,074–16,280	207	68	66	83.3	66	84.8
pVI	16,341–16,970	630	209	218	81.8	218	82.8
Hexon	17,053–19,785	2733	910	910	82.1	910	82.5
Protease	19,786–20,394	609	202	200	77.0	200	76.0
DBP	20,457–21,695c	1239	412	413	72.2	413	72.0
100K	21,710–23,716	2007	668	650	77.9	650	78.5
33K	23,595–23,736, 23,897–24,153	399	132	142	77.2	140	75.7
22K	23,595–23,921	327	106	118	75.9	116	74.0
pVIII	24,153–24,794	642	213	213	85.9	213	84.9
E3 14.7K	24,797–25,174	378	125	131	52.7	131	48.0
U exon	25,191–25,361c	171	56	55	70.9	55	69.0
Fiber	25,360–27,264	1905	634	593	37.6	579	38.5
E4 ORF6/7	27,287–27,514c, 28,217–28,255c	267	88	88	52.2	88	52.2
E4 34K	27,515–28,255	741	246	245	62.4	245	63.2
E4 ORF3	28,258–28,662c	405	134	141	41.0	141	41.0
E4 ORF2	28,674–29,081c	408	135	135	35.0	135	35.0
E4 unknown2	29,164–29,292c	129	42	N.A.	N.A.	N.A.	N.A.
E4 unknown1	29,329–29,556c	228	75	N.A.	N.A.	N.A.	N.A.
ITR	30,033–30,134	102	N.A.	N.A.	N.A.	N.A.	N.A.

* A lowercase “c” indicates that genes are encoded by the complementary strand; ^†^ N.A. indicates not applicable.

**Table 3:** Detection of bat-derived adenoviruses from *E. helvum* kidney DNA.

Year	Sample ID	Location	No. of Samples		
			Total	Positive	Positive Rate (%)
2010	ZFB10-01–ZFB10-47	Kasanka National Park	47	0	0
	ZFB10-48–ZFB10-52	Ndola	4	0	0
2011	ZFB11-01–ZFB11-38	Ndola	38	0	0
	ZFB11-39–ZFB11-95	Kasanka National Park	57	8	14.04
2012	ZFB12-01–ZFB12-60	Ndola	60	0	0
	ZFB12-61–ZFB12-110 *	Kasanka National Park	49	0	0
2013	ZFB13-01–ZFB13-76	Ndola	76	0	0
	ZFB13-77–ZFB13-111 ^†^	Kasanka National Park	34	0	0
Total			365	8	2.19

* A kidney sample from ZFB12-97 was not available for PCR screening; ^†^ A kidney sample from ZFB13-93 was not available for PCR screening.

## Supplementary Materials

The following are available online at www.mdpi.com/1999-4915/9/12/371/s1, Figure S1: Global genome pairwise comparison of genome homology between EhAdV 06-106 and BtAdVs WIV17/18 using mVISTA, Table S1: Adenoviral genomes used in Figure 3.
