# Supplementary material for: Characterization of a Novel Bat Adenovirus Isolated from Straw-Colored Fruit Bat (Eidolon helvum)
Source: Viruses. 2017 Dec 4;9(12):371. doi: 10.3390/v9120371 (PMC5744146; doi:10.3390/v9120371)
Supplement: Supplementary file 1 [file viruses-09-00371-s001.zip › viruses-246341 supplementary/Supplementary_materials/Figure S1 Caption.docx]

**Figure S1.** **Global genome pairwise comparison of genome homology between EhAdV 06-106 and BtAdVs WIV17/18 using mVISTA.** The y-axis indicates 50–100% identities of genome pairs and the polygonal line shows the homology of the corresponding point in the genome. Arrows (E1A, V and fiber) and a solid line (E4 Unit region) indicate the coding sequence region showing lower identities (<50%) in EhAdV 06-106 compared with BtAdVs WIV17 and WIV18.
