# Supplementary material for: Characterization of a Novel Bat Adenovirus Isolated from Straw-Colored Fruit Bat (Eidolon helvum)
Source: Viruses. 2017 Dec 4;9(12):371. doi: 10.3390/v9120371 (PMC5744146; doi:10.3390/v9120371)
Supplement: Supplementary file 1 [file viruses-09-00371-s001.zip › viruses-246341 supplementary/Supplementary_materials/Table_S1.docx]

**Table S1.** Adenoviral genomes used in Figure 3.

| **Virus** | **Abbreviation** | **Accession no.** | **Note** |
| --- | --- | --- | --- |
| Bat adenovirus | BtAdV 250-A | KX871230 | Strain 250-A |
| Bat adenovirus 2 | BtAdV-2 PPV1 | JN252129 | Strain PPV1 |
| Bat adenovirus 3 | BtAdV-3 TJM | GU226970 | Strain TJM |
| Bat adenovirus | BtAdV WIV9 | KT698853 | Strain WIV9 |
| Bat adenovirus | BtAdV WIV10 | KT698854 | Strain WIV10 |
| Bat adenovirus | BtAdV WIV11 | KT698855 | Strain WIV11 |
| Bat adenovirus | BtAdV WIV12 | KT698856 | Strain WIV12 |
| Bat adenovirus | BtAdV WIV13 | KT698852 | Strain WIV13 |
| Bat adenovirus | BtAdV WIV17 | KX961095 | Strain WIV17 |
| Bat adenovirus | BtAdV WIV18 | KX961096 | Strain WIV18 |
| Bovine adenovirus B | BAdV-B | NC_001876 | Strain WBR-1 |
| Bovine adenovirus D | BAdV-D | NC_002685 | Strain THT/62 |
| California sea lion adenovirus | CSLAdV-1 | KJ563221 | Strain Zc11-030 |
| Canine adenovirus type 1 | CAdV-1 | AC_000003 |  |
| Canine adenovirus type 2 | CAdV-2 | AC_000020 |  |
| Equine adenovirus 1 | EAdV-1 | JN418926 | Strain M1 |
| Fowl adenovirus A | FAdV-A | NC_001720 | Strain Phelps |
| Fowl adenovirus D | FAdV-D | NC_000899 | Strain A-2A |
| Frog adenovirus 1 | FrAdV-1 | NC_002501 | Strain ATCC VR-896 |
| Human adenovirus A | HAdV-A | AC_000005 | Human adenovirus type 12 |
| Human adenovirus B1 | HAdV-B1 | NC_011203 | Human adenovirus type 3 |
| Human adenovirus B2 | HAdV-B2 | NC_011202 | Human adenovirus type 11 |
| Human adenovirus C | HAdV-C | NC_001405 | Human adenovirus type 2 |
| Human adenovirus D | HAdV-D | AC_000006 | Human adenovirus type 17 |
| Human adenovirus E | HAdV-E | NC_003266 | Human adenovirus type 4 |
| Human adenovirus F | HAdV-F | NC_001454 | Human adenovirus type 40 |
| Murine adenovirus A | MAdV-A | AC_000012 |  |
| Ovine adenovirus A | OAdV-A | NC_002513 | Bovine adenovirus 2 |
| Ovine adenovirus D | OAdV-D | NC_004037 | Ovine adenovirus 7 |
| Porcine adenovirus A | PoAdV-A | AC_000189 | Porcine adenovirus 3 |
| Porcine adenovirus C | PoAdV-C | NC_002702 | Porcine adenovirus 5 |
| Simian adenovirus B | SAdV-B | KC693021 | Isolate BaAdV-1 |
| Simian adenovirus 3 | SAdV-3 | NC_006144 | Strain ATCC VR-1449 |
| Simian adenovirus 20 | SAdV-20 | NC_020485 | Strain ATCC VR-541 |
| Simian adenovirus 49 | SAdV-49 | NC_015225 | Isolate C24948 |
| Skunk adenovirus 1 | SkAdV-1 | KP238322 | Isolate SkAdV-PB1 |
| Snake adenovirus | SnAdV | NC_009989 | Strain 145/88 |
| Tree shrew adenovirus 1 | TsAdV-1 | AC_000190 |  |
| Turkey adenovirus A | TAdV-A | NC_001958 |  |
