# Supplementary figures and images for: Characterization of a Novel Bat Adenovirus Isolated from Straw-Colored Fruit Bat (Eidolon helvum)
Source: Viruses. 2017 Dec 4;9(12):371. doi: 10.3390/v9120371 (PMC5744146; doi:10.3390/v9120371)

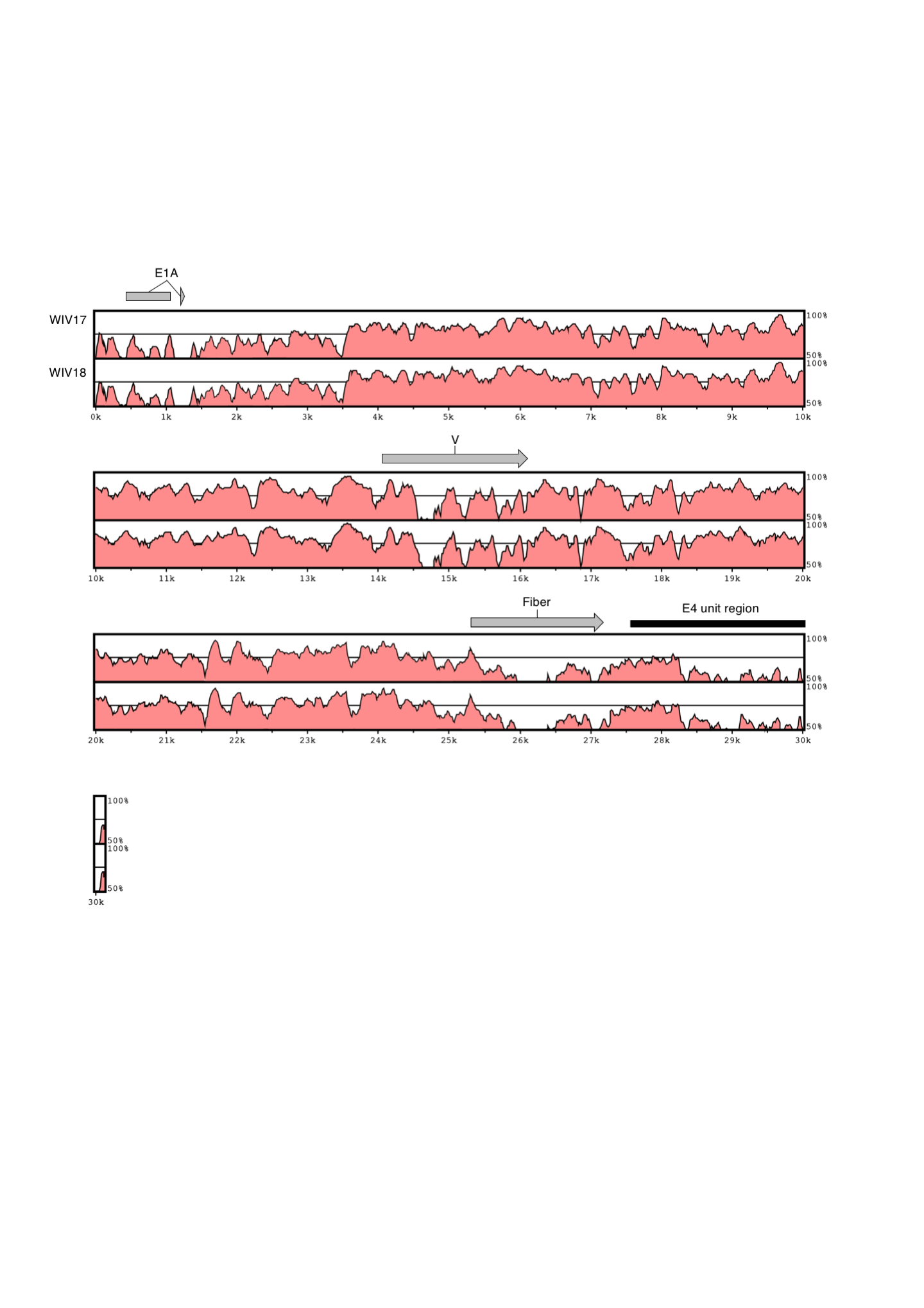

Supplement: Supplementary file 1 [file viruses-09-00371-s001.zip › viruses-246341 supplementary/Supplementary_materials/Figure_S1.tiff]
